# GNSS-Based Non-Negative Absolute Ionosphere Total Electron Content, its Spatial Gradients, Time Derivatives and Differential Code Biases: Bounded-Variable Least-Squares and Taylor Series

**DOI:** 10.3390/s20195702

**Published:** 2020-10-07

**Authors:** Yury Yasyukevich, Anna Mylnikova, Artem Vesnin

**Affiliations:** Institute of Solar-Terrestrial Physics SB RAS, Irkutsk 664033, Russia; manna@iszf.irk.ru (A.M.); artem_vesnin@iszf.irk.ru (A.V.)

**Keywords:** absolute total electron content, bounded-variable least-squares, differential code biases, Taylor series, GPS, GLONASS, ionosphere

## Abstract

Global navigation satellite systems (GNSS) allow estimating total electron content (TEC). However, it is still a problem to calculate absolute ionosphere parameters from GNSS data: negative TEC values could appear, and most of existing algorithms does not enable to estimate TEC spatial gradients and TEC time derivatives. We developed an algorithm to recover the absolute non-negative vertical and slant TEC, its derivatives and its gradients, as well as the GNSS equipment differential code biases (DCBs) by using the Taylor series expansion and bounded-variable least-squares. We termed this algorithm TuRBOTEC. Bounded-variable least-squares fitting ensures non-negative values of both slant TEC and vertical TEC. The second order Taylor series expansion could provide a relevant TEC spatial gradients and TEC time derivatives. The technique validation was performed by using independent experimental data over 2014 and the IRI-2012 and IRI-plas models. As a TEC source we used Madrigal maps, CODE (the Center for Orbit Determination in Europe) global ionosphere maps (GIM), the IONOLAB software, and the SEEMALA-TEC software developed by Dr. Seemala. For the Asian mid-latitudes TuRBOTEC results agree with the GIM and IONOLAB data (root-mean-square was < 3 TECU), but they disagree with the SEEMALA-TEC and Madrigal data (root-mean-square was >10 TECU). About 9% of vertical TECs from the TuRBOTEC estimates exceed (by more than 1 TECU) those from the same algorithm but without constraints. The analysis of TEC spatial gradients showed that as far as 10–15° on latitude, TEC estimation error exceeds 10 TECU. Longitudinal gradients produce smaller error for the same distance. Experimental GLObal Navigation Satellite System (GLONASS) DCB from TuRBOTEC and CODE peaked 15 TECU difference, while GPS DCB agrees. Slant TEC series indicate that the TuRBOTEC data for GLONASS are physically more plausible.

## 1. Introduction

Currently, the global navigation satellite systems (GNSS) enable the study of the ionosphere at any spot on the globe. Such studies are based on dual-frequency phase and pseudorange measurements of the total electron content (TEC) [1]. Since the phase measurements draw a lower level of noise compared to pseudorange measurements, most studies use the phase measurements to determine TEC. There are classical papers [2,3], as well as many up-to-date ones (for example, see [4] and references therein). Lanyi and Roth [2], for the first time, suggested mapping the TEC. They compared, mapped and measured total ionospheric electron content by using global positioning system (GPS) and beacon satellite observation and revealed that the difference between them is less than 1 TECU. Pioneering papers by Calais and Minster [3] showed the efficiency of dual-frequency phase measurements to investigate ionospheric responses to the strong ground displacement associated with earthquakes. Afraimovich et al. [4] presented a number of new results when studying the ionospheric irregularities caused by solar flares, solar eclipses, solar terminators, earthquakes, magnetic storms, rocket launches and tropical cyclones based on GPS/GLObal Navigation Satellite System (GLONASS) data. The studies showed the efficiency of dual-frequency phase measurements. А comprehensive study by Astafyeva et al. [5] showed a high potential of TEC phase measurements to monitor natural hazards. Nesterov and Kunitsyn [6] implemented 4D GNSS radio tomography based on phase measurements. Kunitsyn et al. [7] suggested a new approach to use L1/L5 phase measurements from satellite-based augmentation system. However, when using phase data, there is an ambiguity of phase measurements. For some applied problems, we need not relative, but absolute measurements. Forte and Aquino [8] evaluated the ionospheric effects on low frequency radio astronomy measurements. Afraimovich and Yasukevich [9] suggested an approach based on absolute TEC data for ionospheric correction of radio telescopes and radio interferometers. Ovodenko et al. [10] used absolute GNSS TEC data to correct radar data.

Pseudorange measurements are thought to be absolute, however, they are noisy. For this reason, one often uses pseudorange and phase measurements jointly to eliminate the phase ambiguity. Herewith, there is a bias related to a different time of the signal passage through the channels of a satellite and a receiver. In literature, this error is referred to as differential code biases (DCBs). This error is known to vary systematically, and it may have irregular variations [11]. Mylnikova et al. [12] showed some cases of DCB annual variations caused by receiver/antenna environment conditions. Instability in DCB can be caused by change in grounding [13]. А change in DCB can lead to significant apparent TEC variations [14]. There are some procedures to estimate the DCBs [15,16]. Hong et al. [15] suggested an efficient algorithm to estimate DCB for a network, when one of receiver DCBs is already known. Jin et al. [16] published MatLab code for DCB calculation. They reported an agreement with the global ionospheric map (GIM) data with a mean difference of less than 0.7 ns (~2 TECU) and an RMS of less than 0.4 ns (~1 TECU). Li et al. [17] analyzed triple-frequency combinations and proved that the real satellite DCBs are time-varying. Wang et al. [18] mitigated variations in DCB by separating DCB from ambiguity. As a rule, the DCB estimates are a by-product of estimating the model parameters of TEC measurements. Having the DCB estimated, one can correct the slant TEC series and then use these TEC absolute values in applications.

An important goal is to obtain absolute characteristics reflecting ionospheric conditions. A good way is to have an electron density profile that an incoherent scatter radar or an ionosonde can provide [19,20,21,22]. The cost of such equipment is very high, particularly that of radars. Therefore, the world network of such facilities is limited.

Using the GNSS data can also provide estimates for the electron density profile. This information may be obtained by using 4D spatio-temporal ionospheric tomography suggested by Mitchell and Spencer [23]. GNSS-tomography results agree with ionosphere models and ionosonde data [24]. The tomography algorithms have been developed over the last decades. Additional data (such as ionosonde and Langmuir probes [25]) and new reconstruction algorithms [26] enhance GNSS-tomography. However, these techniques require complicated calculation and large computation resources. Also, for precise measurements, it is necessary to have a very dense network, as is available in Japan or in the United States.

When using the data from sparse networks of stations, a more promising way is to build TEC maps. The International GNSS Service (IGS) Working Group on Ionosphere [27] provides the most common TEC maps. Such global TEC maps are used to analyze the regional or global electron content [28]. For regions with a small number of stations, it makes sense to estimate the vertical TEC over the station.

Currently, there are several methods to estimate the vertical TEC. These methods are based on various TEC models [29,30]. One usually uses the spherical harmonic expansion to build global vertical TEC maps. Estimation of the vertical TEC over the station by using spherical harmonics is not appropriate, it is better to use other expansions. For example, Durmas and Karslioglu [31] use a TEC model based on expansion of basic functions that are the products of univariate B-splines with different scales and variables. There are also methods based on simple polynomial expansion. For the first time, this method was offered by Lanyi and Roth [2] and by Sardón and Zarraoa [32], and then was used by Themens et al. [33], who studied the nature of seasonal and solar cycle bias variability in the polar cap region and showed the influence of user’s choice of shell-height on standard DCB estimation procedure. Also, the Taylor series expansion (as a generalized expansion) of TEC is used as a TEC model; this expansion on space was mentioned by Schaer [34] for regional vertical TEC modeling. We should note that Li et al. [35] mentioned a problem with unstable DCB at some BeiDou satellites and suggested two-step technique to reduce effects of such satellites on overall solution.

Different techniques for TEC estimation do not solve the problem of negative TEC values, especially negative slant TEC on some line of sights. Start and Parker [36] and Waterman [37] solved the mathematical problem for constrained (or bounded-variable) least squares. Zhang et al. [38] used such an approach to improve GIMs. We developed an algorithm to recover the absolute vertical TEC, its gradients, and its time derivatives from a single GNSS station data, as well as slant TEC along all lines of sight and DCB. The procedure uses the Taylor space-and-time expansion and constrained least squares. It provides both non-negative vertical TEC and non-negative slant TEC along all lines of sight. The algorithm can use data from MEO GPS/GLONASS/Galileo/BeiDou satellites. A combined solution can also use the GEO satellite data. However, here, we show only the GPS and GLONASS data. We termed the algorithm TuRBOTEC: TayloR-series and bounded-variable-least-squares-based iOnosphere TEC.

## 2. Data and Background

**Figure 1:**
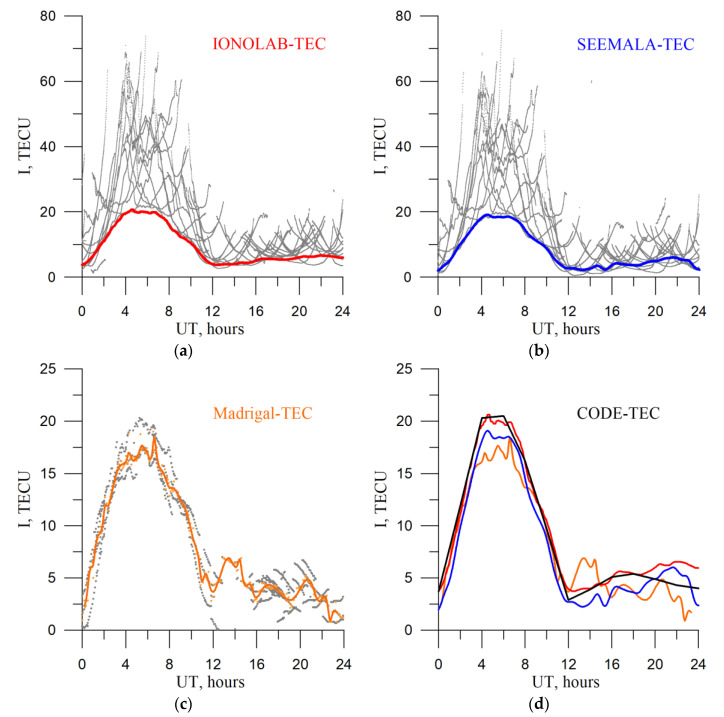
IONOLAB TEC (**a**), SEEMALA-TEC (**b**), Madrigal TEC (**c**). Grey dots on panels (**a**,**b**) show absolute slant TEC from different satellites. Grey dots on panel (**c**) show the vertical TEC for different cells (50°–54°N, 102°–106°E). Orange dots show the median along the 5° × 5° region, while the orange line shows the spline interpolation regarded as the absolute vertical TEC. Panel (**d**) shows all the mentioned vertical TECs along with the TEC from CODE GIMs (black line). The data are for 1 January 2014.

We use the GNSS data from the IGS network [39] as the TuRBOTEC input data. We use the data from three stations: IRKJ, NTUS, and THU2. Table 1 shows the stations’ coordinates: geographic latitude (Lat), geographic longitude (Lon), geomagnetic latitude (MLat), geomagnetic longitude (MLon). The coefficient α is a parameter for the modified single-layer model mapping function (see below). To compare with the obtained results, we used data from the IONOLAB software (http://www.ionolab.org/) [40] and software by Seemala [41], as well as data from ionospheric maps: Madrigal TEC [42] and CODE GIM [34]. It is free-to-use software and data. Figure 1 shows TEC from different techniques for the IRKJ region (52° N, 104° E). The upper panels display the IONOLAB-TEC (left, red line for vertical TEC) and SEEMALA-TEC (right, blue line for vertical TEC). The left bottom panel shows Madrigal TEC (orange line for vertical TEC). The right bottom panel shows TEC from all the above methods and TEC from CODE GIM.

The IONOLAB software is based on a single-station solution [40]. The software uses only GPS data. The input is a pseudorange slant TEC corrected by CODE DCB. Slant TEC is projected to the local zenith to obtain the vertical TEC. Actually it is a zero order expansion. The least-squares method is used to obtain TEC estimates. Our experience showed the problem when negative or zero values appear: negative TEC values result in rejecting data for the whole day.

Seemala developed a software for TEC calculations (referred to as SEEMALA-TEC) [41] based on a similar approach as that in the IONOLAB. However, it uses phase TEC leveled by pseudorange TEC. The vertical TEC is estimated as a mean from all satellites’ vertical TECs. The software can estimate its own DCB, but our experience showed that it is better to use CODE DCB.

Madrigal TEC is also based on projection of slant TEC to vertical TEC, but for different cells (1° × 1°) on the map [42]. They use a novel 3-steps technique to estimate DCB. Averaging data in cells improves the data. However, in the regions, where there are few stations, it is difficult to have continuous and reliable data. Thus, we use a 5° × 5° region around a GNSS station and choose the median value as a Madrigal vertical TEC. Spline is used to smooth median TEC series.

Global ionosphere maps are used for a long time to monitor and model the ionosphere [27]. We used CODE GIMs [30] that are based on spherical harmonics and modified single layer mapping function. We also used JPL GIMs based on multi-layer model. The GIM time resolution was 2 h for the analyzed data. We performed an algorithm operation simulation based on the international reference ionosphere model IRI-2012 [43] and the IRI-plas model [44] as a test.

## 3. TuRBOTEC Algorithm

To obtain absolute TEC, one estimates the preset measurement model parameters by using experimental data. As a rule, one uses the following model of TEC:

*I_M_* = *I_S_* + *I_DCB_* = *I_V_∙S + I_DCB_* (1)

where *I_M_* is the model (expected) value of the recorded slant TEC, *I*s is the slant TEC along the line of sight without a differential code bias, *I_V_* is the vertical TEC estimate at a point corresponds with the measurement (ionospheric pierce point), *S* is the function that converts the slant TEC into the vertical one (mapping function), and *I_DCB_* is an error related to the DCB of the satellite and the receiver.

For geostationary Earth orbit (GEO) satellites it is difficult to estimate DCB (or similar error in single frequency data) from (1), because *S* in Equation (1) is a constant. The corresponding error can be regarded as a TEC offset [45]. However, GEO satellites’ DCB could be estimated along with DCB of medium Earth orbit (MEO) satellites [46].

Ma and Maruyama [47] used de facto the approximation *I_V_=I_V_ (ϕ_0_, l_0_, t_0_)*, where *ϕ_0_*, *l_0_*, and *t_0_* are the station latitude, longitude, and the time, for which the calculation is performed. This implies that all the TEC measurements were supposed to be performed over the station. However, such an approach does not allow one to obtain spatial gradients. Such an approximation is correct in a limited number of cases when the spatial gradients and the time derivative can be neglected. In the most studies, a spatial-temporal expansion of the *I_V_* function is used. The spherical harmonic expansion is often used. Komjathy et al. [48] increased the capacity of GIM technique to simultaneously treat data from more than 1000 dual-frequency GPS receivers. Zhang et al. [49] used the DCB estimation technique based on spherical expansion to evaluate BeiDou DCBs. According to Schaer [34], the Taylor expansion is more appropriate than the spherical harmonic expansion for regional modeling. Schaer [34] mentioned the *I_V_* Taylor series expansion in (1) only on space. In our research, we expand the vertical TEC function *I_V_* (*ϕ, l, t*) into the Taylor series on space and time:

(2) $$I_{V}\left(\phi ,l,t\right)=\overset{\infty }{\underset{m=0}{\sum }}\overset{\infty }{\underset{n=0}{\sum }}\overset{\infty }{\underset{k=0}{\sum }}\frac{{\left(\Delta \phi \right)}^{m}{\left(\Delta l\right)}^{n}{\left(\Delta t\right)}^{k}}{m!n!k!}\frac{\partial^{m+n+k}I_{V}}{\partial \phi^{m}\partial l^{n}\partial t^{k}}$$

where Δ*ϕ*, Δ*l* are the difference between latitude, longitude of the ionospheric pierce point and the point of station (*ϕ_0_, l_0_*), Δ*t* is the difference between time of measurement and *t_0_*.

**Figure 2:**
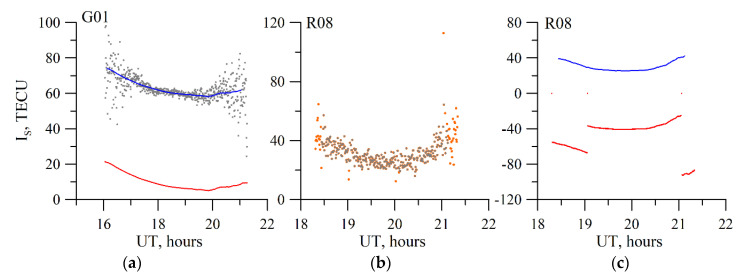
Eliminating the phase ambiguity (**a**), outliers (**b**), and cycle slips (**c**). The panels show the numbers for the corresponding GNSS satellite. Red dots mark the uncorrected phase TEC, blue dots-corrected phase TEC, orange dots-uncorrected pseudorange TEC, grey dots-corrected pseudorange TEC.

We developed the following algorithm to estimate the vertical TEC, the TEC gradients, the time derivative, and DCBs. The algorithm involves:

(1) Calculating TEC based on the pseudorange *I_P_* and phase *I_φ_* measurements. For the analysis, we use the data with elevations greater than 10°.
(2) Dividing the data into continuous samples.
(3) Detecting and eliminating outliers and cycle slips in the TEC data [50] (Figure 2).
(4) Eliminating the phase measurement ambiguity (“leveling”, see Figure 2a):
  $const=\frac{1}{N\sum_{i=1}^{N}S_{i}^{-1}}\cdot \sum_{i=1}^{N}\frac{\left(I_{P}-I_{\varphi }\right)}{S_{i}}$, where *N* is the number of measurements over a continuous interval, *S* is the mapping function (see below). At this state we obtain experimental slant TEC *I_Exp_*.
(5) Estimating DCBs by a simple measurement model and determining the model parameters based on minimizing the model data root-mean-square deviation.

Although in some research the zero-order expansion of (2) is used (only term with *m* = 0, *n* = 0, *k* = 0 in Equation (2)), indeed, it is necessary to substantiate the selection of the expansion order in greater detail. We performed an analysis by using the IRI-2012 model. The TEC slant values, corresponding to real observed elevations and azimuths, as well as the vertical TEC, the time derivative, and the spatial gradients were modeled by latitude and by longitude. Then, we recovered the values of vertical TEC (*I*_V_) from slant measurements by using expansions to the specified order of Taylor-expansion.

At the first step, this recovery was performed based on the zero-order Taylor expansion (2) (leaving term with *m* = 0, *n* = 0, *k* = 0 in (2)). Next, TEC was recovered by using the first-order (leaving terms with *n* + *m* + *k* ≤ 1 in (2)) and second-order (leaving terms with *n* + *m* + *k* ≤ 2 in (2)) expansions.

**Figure 3:**
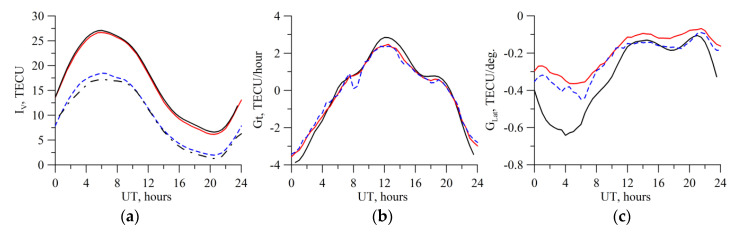
Results of simulating the recovery of the absolute vertical TEC (**a**), the time derivative (**b**), and the latitudinal gradient (**c**) for 10 April 2012. Red solid line indicates values (vertical TEC, time derivative, and spatial gradient) recovered by using the second order *I_V_* expansion; blue dotted line indicates values recovered by using the first order *I_V_* expansion, dot-and-dash black line shows values recovered by using the zero order *I_V_* expansion). The solid black exhibits the IRI-2012 data.

Figure 3 provides the results for recovery of the vertical TEC, the time derivative, and the latitudinal gradient. The solid black line denotes the model values on the plots. The recovered parameters obtained based on of the zero order are denoted by the black dash-dotted line, those based on the first order―by the blue dotted line, and those based on the second order―by the red solid line. From the plots, one can see that using the second order of the *I_V_* expansion into the Taylor series, viz., using of time derivatives and square gradients on space, considerably improves the absolute vertical TEC recovery accuracy.

Meanwhile, using the second order provides a sufficient accuracy to be able to neglect higher orders. Therefore, we use the expansion *I_V_* into the Taylor series up to the second order:

(3) $$I_{M}=S_{j}^{i}\left[\begin{array}{l} I_{V}\left(\phi_{0},l_{0},t_{0}\right)+G_{\phi }\Delta \phi_{j}^{i}+G_{q\_\phi }{\left(\Delta \phi_{j}^{i}\right)}^{2}+\\ +G_{l}\Delta l_{j}^{i}+G_{q\_l}{\left(\Delta l_{j}^{i}\right)}^{2}+G_{t}\Delta t_{j}^{i}+G_{q\_t}{\left(\Delta t_{j}^{i}\right)}^{2} \end{array}\right]+I_{DCB,j},$$

where *I_V_* is the absolute vertical TEC value, Δ*ϕ (*Δ*l)* is the latitude (longitude) difference between the ionospheric point coordinate *ϕ*(*l*) and that of the station *ϕ_0_*(*l_0_*), Δ*t* is the difference between the measurement time *t* and the time *t_0_* for which the calculation is performed. Further, *G_ϕ_ = ∂I_V_/∂ϕ, G_l_ = ∂I_V_/∂l*, *G_q_ϕ_ = ∂^2^I_V_/∂ϕ^2^*, and *G_q_l_ = ∂^2^I_V_/∂l^2^* are the linear and quadratic spatial TEC gradients, and *G_t_ = ∂I_V_/∂t* and *G_q_t_ = ∂^2^I_V_/∂t^2^* are the first and second time derivatives. Equation (3) represents the second-order Taylor series expsion of (1). We neglect the mixed derivatives.

In the literature different mapping functions are used. Selecting the most correct one is a challenge. In most papers, one uses the one-layer approximation of the ionosphere as a thin layer. This approach was suggested by Klobuchar [51]. Hernández-Pajares et al. [52] used the two-layer approximation of the ionosphere. Based on a tomographic procedure, Lyu et al. [53] proposed a climatological mapping function―the Barcelona Ionospheric Mapping Function. There is another approach: Schüler and Oladipo [54] suggested one use the NeQuick model instead of the thin layer approximation to convert the slant TEC into the vertical one. However, such an approach did not provide an essential accuracy increase [54]. We use the modified single-layer model mapping function, and coefficient *α* is introduced to more correctly account for the ionospheric layer height [34]:

(4) $$S_{j}^{i}={\left[cos\left\{\arcsin\left(\frac{R_{E}}{R_{E}+h_{max}}\cdot \sin\left[\alpha \left(90-\theta_{j}^{i}\right)\right]\right)\right\}\right]}^{-1},$$

where *R_E_ =* 6371 km is the Earth radius, *h_max_ =* 450 km is the ionospheric point height, and *α* is the correcting coefficient.

**Figure 4:**
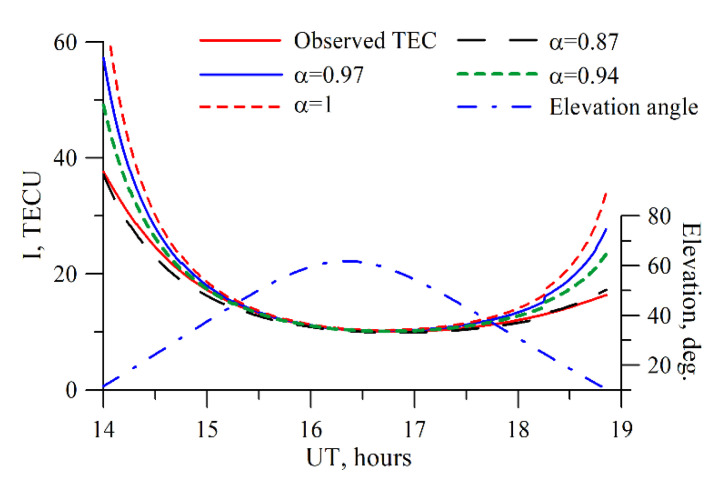
TEC dependence on the elevation for different *α*. The simulation used the IRI-2012 model. The red solid line shows the IRI-2012 slant TEC data; the red dotted line exhibits the slant TEC converted from the vertical one by using *α*=1; the blue solid line shows the slant TEC converted from the vertical one by using *α* = 0.97; the green dotted line shows the same, but with the use of *α* = 0.94; the black dotted line exhibits the same, but with the use of *α* 0.87. The blue dot-and-dash line shows the elevation of the satellite.

Figure 4 presents the slant TEC dependence, *I_S_*, on the elevation when converting *I_S_ = S∙I_V_* for different values *α*. Simulation used the IRI-2012. By its physical implication, the coefficient *α* adjusts a sharp non-physical TEC growth at low elevations, less than 30°.

It is worth noting that due to the ionosphere peculiarities, the coefficient *α* should be latitude-dependent. Also, the *α* parameter should affect the ionosphere disturbance level that may dramatically vary the ionization global distribution [55]. We estimated the *α* values for several points on the earth surface. Table 1 presents the results.

We obtain the set of equations by minimizing the functional *U* = ∑*U^k^* (5) for the set of selected instant *t^k^*_,_ for which we estimate the parameters. For computation, we represent (5) in the form (7):

(5) $$U^{k}=\sum_{j=1}^{N^{k}}\sum_{i=1}^{N_{j}^{k}}\omega^{k,}{}_{j}^{i}{\left(I_{M}{}_{j}^{i}-I_{Exp}{}_{j}^{i}\right)}^{2},$$

(6) $$\omega^{k,}{}_{j}^{i}\equiv \omega^{k}\left(t_{j}^{i}\right)=\Theta \left(t^{k}-t_{j}^{i}+\Delta t\right)\Theta \left(t_{j}^{i}+\Delta t-t^{k}\right)\frac{1}{S_{j}^{i}}{\left[1+{\left(\frac{\Delta t_{j}^{i,k}}{1\;hour}\right)}^{2}\right]}^{-1},$$

**Figure 5:**
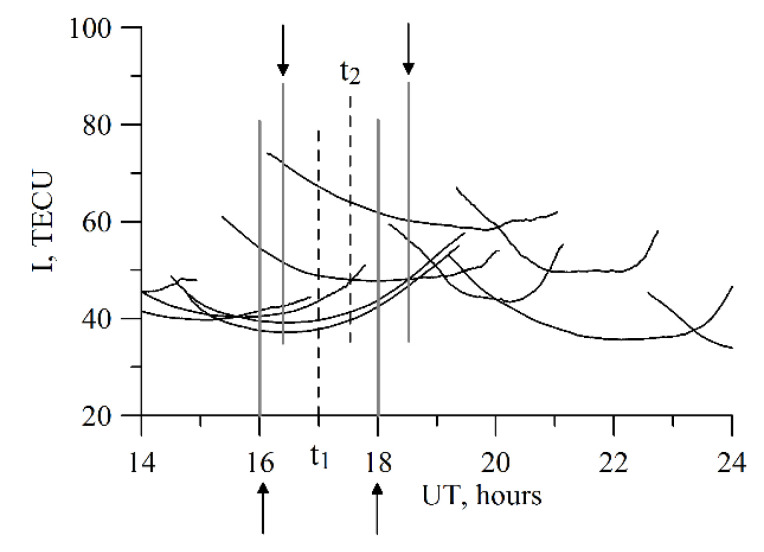
Contribution of measurements from individual satellites to the estimates at different instants.

*U* = ∑*U*^k^ = ||Ax –b ||^2^ -> min, (7)

where *I_Exp_* is the experimental phase TEC measurements with the eliminated phase ambiguity obtained after Stage 4 of the algorithm, Θ is the Heaviside step function, $t_{j}^{i}$ is the *i*th instant of measurement for the *j*th satellite, $\Delta t_{j}^{i,k}$ is the time difference between the current measurement $t_{j}^{i}$ and the time *t^k^*, for which calculating is performed, and Δ*t* = 1 h is the maximal time difference, for which the data are still used for analysis (Figure 5 shows which measurements affect the *t^k^*-estimate). The *1/S* factor in Equation (6) causes the measurements at high elevations to produce the greatest contribution. For each time instant *k*, we have 7∙*J* + *N* variables (*I^k^_V_*, *G*^k^ *_ϕ_, G*^k^*_l_, G*^k^*_q _ ϕ_*, *G*^k^*_q_l_*, *G*^k^*_t_*, *G*^k^*_q_t_,* and *I_DCB, j_*) in Equation (5), where *J* is the number of instants over the investigated interval, for which calculation is performed, and *N* is the number of the satellites observed.

After that, minimization should be applied, which is based on the least square technique. A typical problem for TEC estimation is emerging negative or zero values. For GIM data, this leads to zero values in some GIM cells. Actually, the solution for such problems was suggested a long time ago. We need to restrict the estimated values [36,37]. First results to apply restriction was used to improve GIMs [38]. For our task, we need to obtain both non-negative vertical TEC and non-negative slant TEC. The slant TEC after DCB removal should at least exceed some value *C*. So we introduce the next boundaries:

*I*_V_(*t*_0_) > *C*, ∀*t*_0_ (*I*_DCB_)*_j_* < (*I*_Exp_)*_min_*,_j_ – *C*, ∀ satellite j (8)

where *C* is a non-negative value of minimal TEC, which can be observed in principle. We chose *C* = 0.5 TECU.

We used the Python library scipy.optimize.lsq_linear based on algorithm suggested by Start and Parker [36]. The main steps of the algorithm are as follows:

(1) The algorithm first computes the usual least-squares solution. This solution is returned as optimal, if it lies within the bounds. If not, the algorithm finds all variables within the bounds (free set) and beyond (active set).
(2) At each iteration the algorithm chooses a new variable (which has maximal gradient of the squared objective) to move from the active set to the free set.
(3) New equation system for free set is created where *b* in (7) is changed by active set. Least-squares solution for new equation system contains variables beyond the bounds, the gradient correction is applied to all the free set (see [36] for details).
(4) The iterations continue until all the variables are in the free set.

This algorithm ensures an accurate solution eventually, but may require about *n* iterations for a problem with *n* variables. As a result we obtain non-negative (positive) vertical TEC and slant TEC at all the lines of sight. To obtain robust DCB estimates *I*_DCB_, we calculate the parameters simultaneously for different time instants over 24 h, thus solving a consistent set of Equations (7). The temporal resolution for *t^k^* may vary from 2 h to 5 min.

To analyze the satellite DCB separately, we apply the often-used zero-mean condition [34]:

(9) $$\sum_{i=1}^{N}I_{DCB}^{i}=0,\;$$

where *N* is the number of satellites in the constellation. We applied condition (9) for GPS and GLONASS (Galileo and BeiDou) separately, following Schaer [56].

To simulate the algorithm operation, we used the IRI-2012 model with a set of the International Union of Radio Science (URSI) coefficients, recommended by the URSI and the IRIcorr topside ionosphere profile option [43]. To check the influence of the plasmasphere, we performed calculations based on the IRI-plas model [44]. Currently IRI-plas is a standard for the plasmasphere calculation.

Simulation was performed for a selected real station. For each recorded satellite at each time instant, we calculated the electron density and TEC along the line of sight. Further, we introduced the DCB-related error, as well as random noise to the phase TEC and the pseudorange TEC. This value was 0.01 TECU for the phase TEC. For the pseudorange TEC, the noise value was assigned depending on the elevation: five TECU at *θ* > 60°; 5 + 1·*S*^10^(*θ* + 30) TECU at *θ* < 60°. Also, we introduced outliers for the pseudorange and losses of phase lock for the phase, which could occur with a probability. Thus, at the output, we obtained a series of the phase TEC and the pseudorange TEC corresponding to a certain time instant, elevation, and azimuth. Further, we used these values and calculated ionospheric parameters based on the above algorithm. Finally, the parameters obtained as a result of the algorithm operation were compared with the modeled vertical TEC, time derivatives, and gradients. The obtained DCBs were compared with those specified as the simulation input.

## 4. Technique Validation and Discussion

We validated data based on ionosphere modeling and TEC products from alternative software mentioned in Section 2.

### 4.1. Absolute Total Electron Content

**Figure 6:**
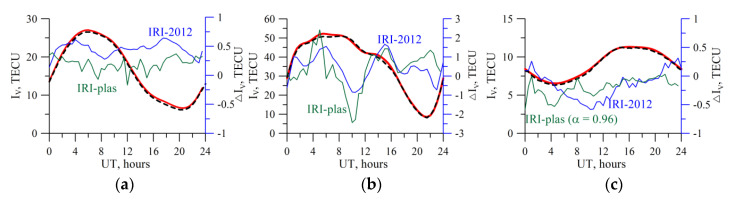
Results of simulating the absolute vertical TEC recovery for different stations as 10 April 2012: (**a**) mid-latitude IRKJ, α = 0.97; (**b**) equatorial NTUS, *α* = 0.87; (**c**) high-latitude THU2, *α* = 0.94. The black dotted line shows the results of the TuRBOTEC operation, the red line exhibits the IRI-2012 vertical TEC data, and the blue line shows differences between the results of the TuRBOTEC operation and the IRI-2012 model. The green line shows the similar differences, but for the IRI-plas simulation. The axis for the blue and green lines are on the right, for the red and black lines on the left.

Figure 6 presents the results of the IRI-2012 simulating the TuRBOTEC algorithm operation (black dotted lines) for different stations: the mid-latitude IRKJ, equatorial NTUS, high-latitude THU2. The results were obtained for the 10 April 2012. One can see good qualitative and quantitative agreement of the vertical TEC recovery results with the IRI-2012 input data (red line). The average deviation for IRKJ by the absolute value is 0.1 TECU, maximal is 0.3 TECU, and RMS being 0.09 TECU. For NTUS, the average deviation is 0.4 TECU, maximal 0.95 TECU, RMS being 0.35 TECU. For THU2, the average deviation is 0.23 TECU, maximal −0.6 TECU, RMS being 0.17 TECU.

The differences between TuRBOTEC simulation and the IRI-plas vertical TEC are shown in green. The results show that TuRBOTEC provides even better performance for modeling based on IRI-plas at mid-latitudes, and comparable performance at low latitudes. Higher difference at high latitudes can be due to using α obtained through the IRI-2012 modeling, so we used *α*=0.96 for THU2. We did not find significant influence of the plasmosphere on TuRBOTEC estimations, except high latitudes, where we should to use another *α* in (4).

The greatest deviation occurs in the equatorial region. This is related to a substantially inhomogeneous ionosphere structure in this region, particularly, during daylight hours. The error is less at high latitudes. Although, as compared with the mid-latitude region, the error is higher. This is related to the absence of satellite observations at high elevations in high latitude regions, and to the dominance of the southward contribution to the total measurement statistics.

**Figure 7:**
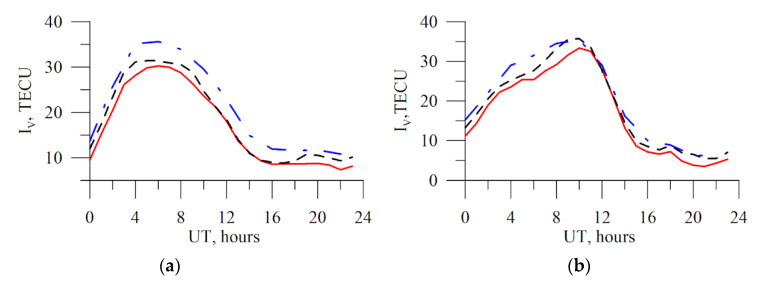
Absolute vertical TEC recovered from real the GPS/GLONASS data for IRKJ (red line). Panel (**a**) is for 5 March 2015 (*Кр_max_* = 2.3), panel (**b**) 17 March 2015 (*Кр_max_* = 7.7). The dot-and-dash blue line presents the JPL data, the black dotted line presents the CODE data, and the red solid line shows the TuRBOTEC values.

Figure 7 presents the results of estimating the vertical TEC for IRKJ by real dual-frequency measurements from GPS and GLONASS (red line). The dot-and-dash blue line and the dotted black line show the data on GIMs from the JPL and CODE laboratories, respectively. The data are presented for the 5 March 2015 quiet conditions (left panel, *Kp_max_* = 2.3, maximal *Kp* for the day) and for the 17 March 2015 magnetic storm (right panel, *Kp_max_* = 7.7).

One can see well that the TEC curves reproduce the diurnal variation similarly, but they can quantitatively differ. Such systematic differences are well-known and were repeatedly addressed in literature [57]. Like discussed earlier, the JPL data, as a rule, surpass the data from other laboratories.

One can see that, at individual instants, synchronous variations with TEC are noted in the CODE and TuRBOTEC data. For example, for the 17 March 2015 strong magnetic storm, one can see a slight increase in the vertical TEC around 18 UT. In general, the estimates for the vertical TEC appear plausible for all the considered cases. Deviations in the data from other laboratories are within the variance interval of different laboratories among themselves. To analyze systematic variances, we built a histogram for the *ΔI_V_* difference distribution between the vertical TEC data in the Irkutsk region.

**Figure 8:**
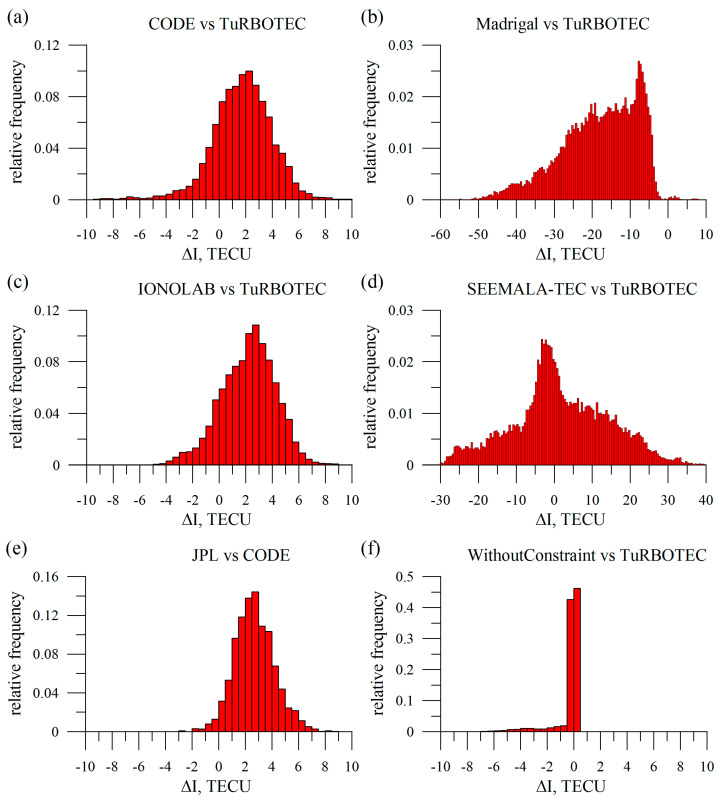
Normalized *ΔI_V_* histograms (difference between *I_V_* values from alternative and TuRBOTEC data). (**a**) GIM CODE vs. TuRBOTEC; (**b**) Madrigal vs. TuRBOTEC; (**c**) IONOLAB vs. TuRBOTEC; (**d**) SEEMALA-TEC vs. TuRBOTEC. Panel (**e**) shows the distribution between the difference in JPL GIM TEC and CODE GIM TEC; panel (**f**) compares techniques with (TuRBOTEC) and without constraint. The data are for the IRKJ over 2014.

Figure 8 shows distribution of differences between the vertical TEC from alternative and TuRBOTEC data. Data for IRKJ station over 2014. Panel (a) shows GIM CODE vs. TuRBOTEC; (b) Madrigal vs. TuRBOTEC; (c) IONOLAB vs. TuRBOTEC; (d) SEEMALA-TEC vs. TuRBOTEC. Panel (e) shows the distribution between the difference in JPL GIM TEC and CODE GIM TEC. (JPL vs. CODE)-distribution (e) features similar against (CODE vs. TuRBOTEC) or (IONOLAB vs. TuRBOTEC) root-mean-squares, that are 1.5 TECU vs. 1.7 or 2.1 TECU, and similar mean - ~2.7 TECU vs. 2.3 or 2.1 TECU. The Madrigal data and the SEEMALA-TEC data show a high discrepancy with the TuRBOTEC: the root-mean-squares were 10–12 TECU. Panel (f) compares the techniques with (TuRBOTEC) and without constraint. It shows that most vertical TECs are the same with and without constraint. However, about 9% of vertical TECs from the TuRBOTEC estimates exceed (by more than 1 TECU) those from the same algorithm but without constraints.

The vertical TEC from the previous version of the suggested technique (without constrains and several other issues) was experimentally checked in [58,59]. Those results also show relevant vertical TEC dynamics during space weather events.

### 4.2. Spatial Gradients. Accuracy of Determining TEC at a Growing Distance from a Station

**Figure 9:**
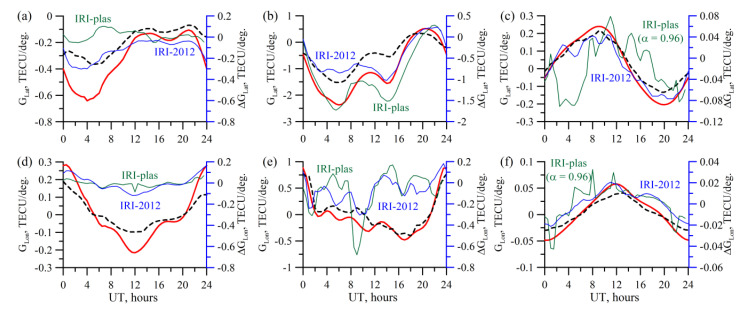
Simulation for the TEC latitude (**a–c**) and longitude (**d–f**) gradients recovery for IRKJ (**a**,**d**), NTUS (**b**,**e**), and THU2 (**c**,**f**). The black dotted line presents the results of the TuRBOTEC operation; the red line exhibits the IRI-2012 TEC gradient data, and the blue line shows the difference between the results of the TuRBOTEC operation and those from the IRI-2012 model. The green line shows the similar differences, but for the IRI-plas simulation. The axis for the blue and green lines are on the right, for the red and black lines on the left.

Figure 9 presents the TEC latitude and longitude gradients for IRKJ, NTUS, and THU2. The data were obtained as a result of simulation based on the IRI-2012. The initial measurements are the same, as those for Figure 6. The parameters presented in the figure are a solution for a consistent set of Equations (7). The black dotted line marks the results of the TuRBOTEC operation; the red line shows the vertical TEC data and gradients from IRI-2012 model.

Absolute gradients differ from <0.1 TECU/deg. for longitude gradients in the high-latitude regions to 2.5 TECU/deg. for latitude gradients in the equatorial regions during the equatorial anomaly evolution.

The IRI-2012 gradients and their TuRBOTEC estimates are qualitatively similar. However, as compared with the vertical TEC recovery accuracy, the accuracy of determining the spatial gradients is substantially lower. Notably, for equatorial stations, the divergence in the latitude gradient may reach 1 TECU/deg. during the equatorial anomaly evolution. This is related to that model (3) involves *S* function that converts the slant TEC into the vertical TEC. In the equatorial anomaly region, such an approximation works worst of all, and, whereas the vertical TEC estimate is quite reasonable (see Figure 6), the spatial gradient estimate may not be satisfactory.

**Figure 10:**
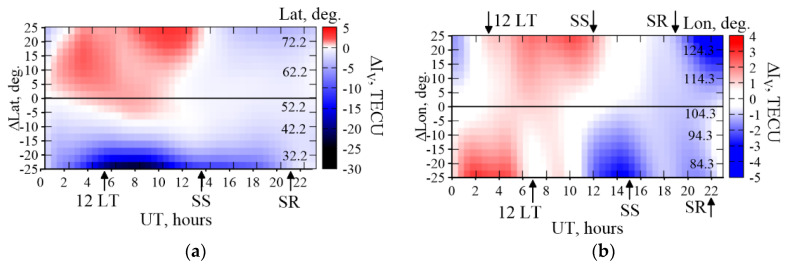
Errors in TEC estimations at different UT for various distances from IRKJ. Δ*I*_V_, is the difference between the model-specified values and the model-recovered data, panel (**a**) and panel (**b**) are for the distance from the station by latitude and longitude, respectively. The IRI-2012 simulation as of 10 April 2012 for the IRKJ geometry.

When estimating the vertical TEC at a growing distance from a station, this leads to an error. In Figure 10, we present the value of this error at different UT for various distances from a station. The figure presents the simulation data for the mid-latitude IRKJ station. Panel (a) shows the error when moving away from the station along the latitude; panel (b) does the same when moving away from the station along the longitude. The arrowlets mark the local midday and the solar terminator time at 200 km: SR is the morning (sunrise) terminator, SS being the night (sunset) one. On panel (b), the time of local midday and the terminator is marked for the longitude extremities: for 124.3° E (arrowlets at the plot top) and for 84.3° E (arrowlets at the plot bottom).

One can see that the latitude errors grow more than when moving at a similar distance by longitude. This may be determined by that the *S* mapping function has an essential latitude dependence. Therefore, at a significant deviation, the vertical TEC value converted from the slant TEC is not precisely determined. Hence, the gradients are not precisely determined, either. It is worth noting that, in this case, this error would grow almost linearly. From Figure 10, one can see that this is not absolutely so. The real and the model latitude gradients have a dramatically spatially inhomogeneous character as one moves away from the station, and, at distances more than 10°–15°, the estimates start to surpass 10 TECU.

### 4.3. TEC Time Derivative

**Figure 11:**
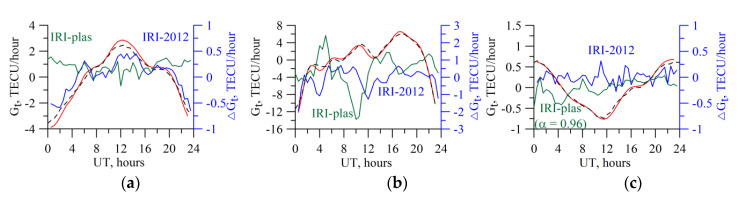
Simulating the TEC time derivative recovery for IRKJ (**a**), NTUS (**b**), and THU2 (**c**) stations. The black dotted line presents the results of the TuRBOTEC operation, the red line exhibits the IRI-2012 TEC time derivative data, and the blue line shows the difference between the results of the TuRBOTEC operation and those from the IRI-2012 model. The green line shows the similar differences, but for the IRI-plas simulation. The axis for the blue and green lines are on the right, for the red and black lines on the left.

Figure 11 shows the results of simulating the algorithm operation to recover the TEC time derivative. We obtained TEC time derivatives when solving the consistent set of equations, whose results are presented in Figure 6 and Figure 9. The designations are the same as those in Figure 6. Similar to the results for the absolute vertical TEC (Figure 6), there is agreement of both general dynamics and the quantitative values of the TEC time derivative. This indicates that such data may be used to efficiently predict the vertical TEC in the station region.

**Figure 12:**
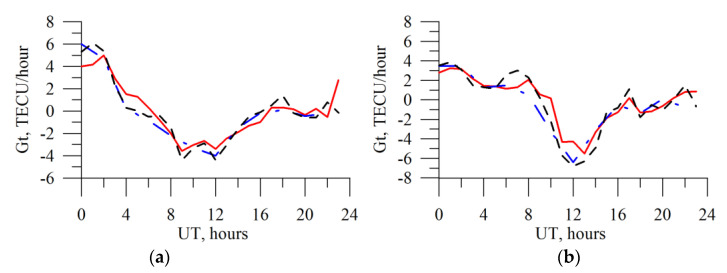
GNSS-based TEC time derivative for IRKJ. (**a**) 5 March 2015 (*Kp_max_* = 2.3); (**b**) 17 March 2015 (*Kp_max_* = 7.7). The blue dot-and-dash line presents the JPL data, the black dotted line denotes the CODE data, and the red solid line displays the TuRBOTEC-obtained values.

Figure 12 presents the results of the TEC time derivative experimental recovery through the TuRBOTEC algorithm from the IRKJ data (red solid curve) and the GIM-obtained time derivative from CODE (black dotted line), from JPL (blue dot-and-dash line) for 5 March 2015, *Kp_max_* = 2.3 (left panel), and for 17 March 2015, *Kp_max_* = 7.7 (right panel). Like for the absolute vertical TEC data (Figure 7), there is qualitative and quantitative agreement with the GIM data, the divergence values from other laboratories are within the divergence interval of different laboratories among themselves.

### 4.4. Differential Code Biases and Absolute Slant TEC

**Figure 13:**
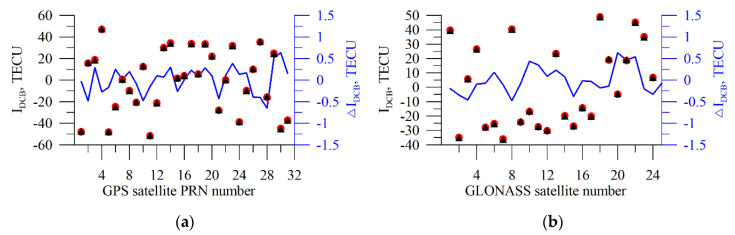
Simulating the DCB recovery from GPS (**a**) and GLONASS (**b**). The black triangles are the initial values; the red circles present the recovered values; the blue line shows the difference between the results of the TuRBOTEC operation and the initial values. The data are in TECU.

Figure 13 presents the modeled results of the DCB (red circles) recovery. The data are in TECU. A random number generator assigned the initial biases (black triangles). Then, there was an IRI-2012 simulation and determining DCBs by a set of integral TEC data through the above algorithm. For simulation, we used the geometry of a real station, IRKJ, as of 10 April 2012.

One can see a good performance of the algorithm both for the GPS data and for the GLONASS data. The maximal error is 0.6 TECU, RMS being 0.3 TECU. The obtained estimates are sufficiently robust both for quiet and for disturbed conditions.

**Figure 14:**
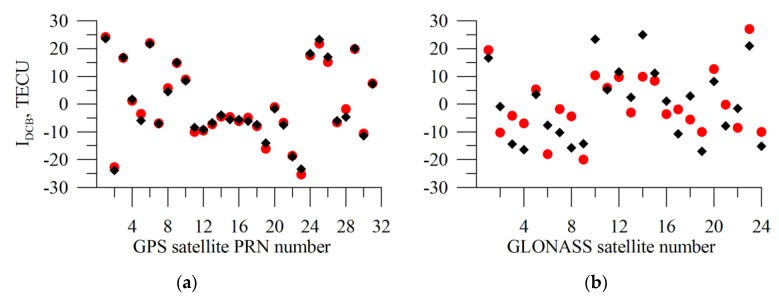
DCBs estimate for GPS (**a**) and GLONASS (**b**) satellites from the IRKJ 1 March 2015 data. The red circles show the results of the TuRBOTEC algorithm operation; the black diamonds present the CODE data.

Figure 14 presents the DCB experimental estimates (in TECU, red circles) obtained from real GPS and GLONASS measurements through the algorithm operation for all the GPS (a) and GLONASS (b) satellites. The estimates were obtained from the IRKJ 1 March 2015 measurements. For comparison, we show the CODE estimates (black diamonds). For the GPS DCBs, the divergence of estimates is sufficiently small. The maximal error for the GPS satellites is 5 TECU, RMS being 2.4 TECU. For the GLONASS satellites, this error is higher. The GLONASS maximal error is 17 TECU, RMS being 8.8 TECU. The results for GPS quite correlate with the estimates by Jin et al. [16]. At the same time, we observe a significant deviation of the CODE and TuRBOTEC estimates for GLONASS. There is no such difference between GPS and GLONASS in simulation. Slant TEC demonstrates non-negative values for GLONASS, when we use TuRBOTEC DCB instead of CODE DCB for GLONASS.

**Figure 15:**
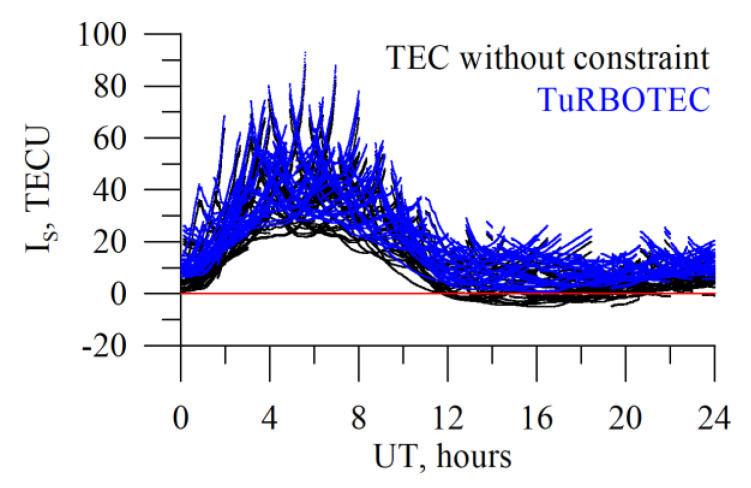
GNSS absolute slant TEC after least squares without constraint (black dots) and TuRBOTEC techniques (blue dots).

Figure 15 shows absolute slant TEC for both techniques with constraint (TuRBOTEC, blue dots) and without (black dots). The data are for 5 January 2014. We show the data for all satellites in view. After least squares without constraint, we note negative TEC values after 12 UT for several satellites. Constraint provides non-negative values for all the satellites.

### 4.5. Influence of Intra-Day DCB Variations

**Figure 16:**
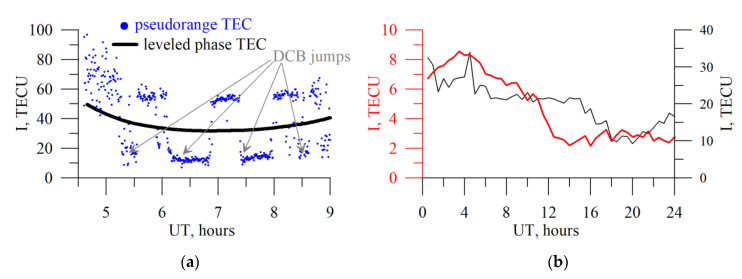
Influence of intra-day DCB variations on pseudorange TEC (**a**, blue dots) and vertical TEC estimates (**b**, black line). Red line on panel (**b**) shows the phase-without-pseudorange solution. The data are for IRKJ 12 July 2009.

Nie et al. [60] revealed that the intra-day DCB variations result in a mis-modeling error of several tenths of TECU. Grounding change could lead to such variations [13]. Figure 16a shows an example of pseudorange TEC that features by rapid step-like changes (jumps). Such huge errors destroy the solution of equation system based on (1). Black line in Figure 16b shows the obtained vertical TEC dynamics without typical daily TEC variation.

We can solve the emerged problem by excluding DCB from Equation (1):

*I_M_* = *I_S_* + *I_DCB_* = *I_V_∙S + I_const_*, (10)

where, *I_const_* is a term corresponding to the phase ambiguity. The term is constant for a continuous TEC series. We change (3) in the same way. Thus, for Equation (7), we exchange *N* variables (where *N* is a number of satellites) by *N’* those (where *N*’ is a number of continuous series). This improves the solution (see red line in Figure 16b), but does not provide DCB anymore. It is the users, who should choose, if they need DCB, and if the pseudorange data quality is sufficient. We would note additionally, that short series should be removed from the treated data.

## 5. Conclusions

We have developed an algorithm to recover the absolute TEC, its gradients, its time derivative, and DCBs. The procedure is based on the space-and-time Taylor expansion and bounded-variable least squares. We termed it TayloR-series and Bounded-variable-least-squares based iOnosphere TEC (TuRBOTEC). We simulated the algorithm operation by using the IRI-2012 and the IRI-plas models. The absolute TEC values recovered through the developed algorithm were established to agree (qualitatively and quantitatively) with the IRI-2012 model-set values. The mean standard deviation for the mid-latitude IRKJ station is 0.09 TECU, for the equatorial NTUS is 0.35 TECU, for the high-latitude THU2 is 0.17 TECU. We did not find significant influence of the plasmosphere on TuRBOTEC estimations, except for high latitudes, where we should use another α for the mapping function. About 9% of experimental vertical TECs from the TuRBOTEC estimates exceed (by more than one TECU) those from the same algorithm but without constraints.

The recovered values of the TEC spatial gradients and of the TEC time derivative agree qualitatively with the model-set values. Also, we studied the accuracy of TEC estimates by means of the latitudinal and longitudinal gradients, for the ionosphere at a distance from a station. The latitude errors were established to grow more dramatically, than those of longitude. This could happen, because the *S* mapping function has an essential latitude dependence. Therefore, at a significant distance, the vertical TEC value converted from the slant TEC is not precisely determined. Hence, the gradients are not precisely determined, either. One should note that, in this case, this error would grow almost linearly, but this is not absolutely so. The real (including the model ones) latitude gradients have a considerably spatially non-uniform character at a distance from a station, and, at distances more than 10°–15°, the TEC estimates based on gradients, start to surpass 10 TECU.

The DCB values obtained through the developed algorithm for GPS satellites agree with the GIM and CODE data, but, for the GLONASS DCB values, the deviation from the CODE data is up to 17 TECU. At the same time, the recovered DCBs (at the IRI-2012 simulation) agree well with the initial data. At large errors in determining DCBs after correcting the slant TEC series, one may observe negative unphysical TEC values.

The developed software may be used to calculate the vertical TEC from the local networks or to locally update ionosphere models [61]. The vertical TEC obtained through this procedure generally agrees with the TEC from the IONOLAB and CODE GIM. The TuRBOTEC data disagree with the Madrigal for a region with few stations and with the SEEMALA-TEC data.

## Figures and Tables

**Table 1:** Position of GNSS stations.

Station	Lat, °	Lon, °	MLat, °	MLon, °	*α*
IRKJ	52.2	104.3	47.7	178.3	0.97
NTUS	1.3	103.7	−7.2	176.3	0.87
THU2	76.5	291.2	83.8	27.1	0.94
